# Synthesis, Characterization, Crystal Structure and Antibacterial Activities of Transition Metal(II) Complexes of the Schiff Base 2-[(4-Methylphenylimino)methyl]-6-methoxyphenol

**DOI:** 10.3390/molecules14051747

**Published:** 2009-05-07

**Authors:** Yu-Ye Yu, Hui-Duo Xian, Jian-Feng Liu, Guo-Liang Zhao

**Affiliations:** 1 Jinhua College of Profession & Technology, Jinhua 321017, P. R. China; 2 Zhejiang Key Laboratory for Reactive Chemistry on Solid Surfaces, Institute of Physical Chemistry, College of Chemistry and Life Sciences, Zhejiang Normal University, Jinhua, 321004, P. R. China; E-mails: hdxian@163.com (H-D.X.); liujianfeng23@163.com (J-F.L.); sky53@zunu.cn (G-L.Z.)

**Keywords:** Transition metal(II) complex, crystal structure, Schiff base, antibacterial activity

## Abstract

Five transition metal(II) complexes, [ML_2_Cl_2_] **1~5**, were synthesized from the reaction of MCl_2_·nH_2_O (M = Mn, Co, Ni, Cu, Cd) and the Schiff base ligand 2-[(4-methylphenylimino)methyl]-6-methoxyphenol (C_15_H_15_NO_2_, **L**), obtained by condensation of *o*-vanillin (2-hydroxy-3-methoxybenzaldehyde) with *p*-toluidine. They were characterized by elemental analysis, molar conductance, FT-IR spectra, thermal analysis. The structure of complex **1** was determined by single-crystal X-ray diffraction. Its crystal structure is of monoclinic system, space group P2_1_/c with a = 9.0111(18) Å, b = 11.222(2) Å, c =28.130 (6) Å, α = 90 º, β = 92.29(3) º, γ = 90 º, V = 2867.6(10) Å^3^, Z = 4. The Mn atom is six-coordinate and displays distorted octahedral geometry. The Schiff base ligand and its complexes have been tested *in vitro* to evaluate their antibacterial activity against bacteria, viz., *Escherichia coli*, *Staphylococcus aureus* and *Bacillus subtilis*. It has been found that the complexes have higher activity than the corresponding free Schiff base ligand against the same bacteria.

## 1. Introduction

Transition metals are necessary for our life, especially Mn, Co and Zn. Manganese is a component of nucleic acids, and can accelerate the synthesis of cholesterol. *o*-Vanillin is a natural aldehyde found in *Andropogen nardus*. It is used to treat bellyaches, and also in spicery [1,2]. Schiff base ligands which usually contain O and N donor atoms have played an important role in coordination chemistry since the late 19^th^ century. Metal complexes with these ligands are becoming increasingly important as biochemical, analytical and antimicrobial reagents, in the design of molecular ferromagnets, in materials chemistry and so on [3,4,5,6,7,8,9,10,11]. We have previously reported the synthesis and antibacterial activities of Zn and Mn Schiff base complexes [12,13]; as an extension of this work, we now report five new Mn(II), Co(II), Ni(II), Cu(II) and Cd(II) complexes of the Schiff base 2-[(4-methylphenylimino)methyl]-6-methoxyphenol, derived from *o*-vanillin and *p*-toluidine.

## 2. Results and Discussion

### 2.1. Elemental analysis, molar conductance

The compositions of the complexes are summarized in Table 1. The C, H, N and M contents (both theoretically calculated values and actual values) are in accordance with the formula ML_2_Cl_2_ indicating that the Schiff base ligand is neutral. This can be explained by the absence of any deprotonating agent during the synthesis. Complexes in which a Schiff base coordinates as a neutral ligand are still rare. Recent studies [14,15] mention complexes of transition metal (II) ions in which the Schiff base coordinates in this unusual structural form via the phenolic hydroxy oxygen atom and the nitrogen atom remains uncoordinated. Their molar conductance values in DMF solution lie in the range of 11~18 S·cm^2^·mol^-1^, as expected for non-electrolytes [16].

**Table 1:** Elemental analysis, molar conductance data of ligand and complexes.

Compound	m.p./ ^◦^C	Color	Elemental analysis / %*				∧_M_ /S·cm^2^·mol^-1^
			C	H	N	M	
**L**	~100	Orange red	74.59 (74.66)	6.30 (6.17)	5.77 (5.81)		2
MnL_2_Cl_2_ (**1**)	~260 (dec)	Red	58.97 (59.22)	4.93 (4.97)	5.67 (5.62)	8.89 (9.03)	18
CoL_2_Cl_2_ (**2**)	~218 (dec)	Red	58.89 (58.83)	4.92 (4.94)	4.56 (4.58)	9.75 (9.62)	18
NiL_2_Cl_2_ (**3**)	~237 (dec)	Orange yellow	58.80 (58.86)	4.88 (4.94)	4.55 (4.58)	9.55 (9.59)	18
CuL_2_Cl_2_ (**4**)	~197 (dec)	Brown	58.35 (58.39)	4.86 (4.90)	4.56 (4.54)	10.22 (10.30)	17
CdL_2_Cl_2_ (**5**)	~286 (dec)	Red	53.93 (54.11)	4.47 (4.54)	4.18 (4.21)	16.53 (16.88)	11

* Note: values in parentheses are the calculated ones.

### 2.2. IR spectra

The IR spectra of the complexes are summarized in Table 2. The broad absorption band at 3,468 cm^-1^ is attributed to the hydroxyl group of the free ligand. In complexes the H atom of the Schiff base ligand has a tendency to migrate to the azomethine N atom *via* N−H···O intramolecular hydrogen bonding and its absorption band appears at 3,445 – 3,449 cm^-1^, showing coordination of oxygen atom of the phenolic hydroxyl with the central M(II), ion as reported in the literature [17]. The shift of the C–O (Ph-OH) stretching vibration from 1,257 cm^-1^ to 1,237 cm^-1^~1,243 cm^-1^ also supports the coordination of oxygen atoms, so we conclude that coordination bonds were formed between the metal ion and the oxygen atoms of the phenol hydroxyl and the methoxy group [7]. However, a strong band in the free Schiff base ligand occurring at 1,614 cm^-1^, attributed to C=N stretching, is found shifted to higher frequency (1,637 cm^-1^~1,644 cm^-1)^, although the azomethine nitrogen atom was regarded as a non M(II)-coordinating atom with [14,15]. A new band at 494 cm^-1^~501 cm^-1^ attributed to M–O stretching vibration also appears, whereas no such band was apparent in the ligand [18].

**Table 2:** Values of IR spectra for the ligand and complexes (cm^-1^).

Compound	υ_OH_	υ_C=N_	υ_C-O _	υ_M-O_
**L**	3,468 (w)	1,614 (s )	1,257 (s )	
**1**	3,445 (m)	1,638 (s )	1,237 (s )	497 (w )
**2**	3,449 (m)	1,641 (s )	1,243 (s )	501 (w )
**3**	3,447 (m)	1,643 (s )	1,238 (s )	495 (w )
**4**	3,448 (w )	1,644 (s )	1,237 (s )	494 (w )
**5**	3,449 (m )	1,637 (s )	1,241 (s )	496 (w )

* Note: s: strong, m: middle, w: weak

### 2.3. Crystal structure

**Figure 1:**
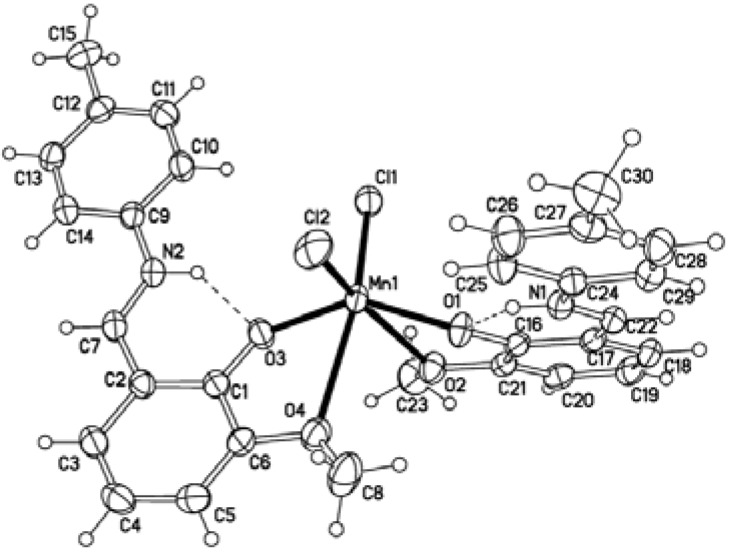
Molecular structure of the complex **1** (probability of ellipsoid is 30%). Intramolecular hydrogen bonds are depicted as dashed lines.

[MnL_2_Cl_2_] (**1**): Single crystal X-ray diffraction analysis reveals that complex **1** contains of one Mn(II) atom, two **L** ligands and two Cl ions (Figure 1). In the [MnL_2_Cl_2_] unit, the Mn1 atom is six-coordinate by four O atoms of the **L** ligand 9Mn–O distances in the range of 2.0771(15)–2.6036(17) Å) and two Cl ions (Mn–Cl distances of 2.3921(9) and 2.4233(9) Å respectively), and displays distorted octahedral geometry. There is a weak interaction between Mn(II) and the O2, O4 atoms with Mn–O distance of 2.5228(16) and 2.6036(17) Å. The mean Mn–O(O2,O4) bond distance in **1** is longer than that in the manganese(II) complex [Mn(C_8_H_7_O_3_)_2_(H_2_O)_2_] (2.3506 Å) [19], but shorter than the Mn–O van der Waals radius (3.37 Å), so we know that there exists a weak interaction between Mn(II) and the O2, O4 atoms. The dihedral angles defined by the two phenyl rings of the same L ligand are of 3.113(66)° and 3.040(68)°, so the phenyl rings are almost parallel to each other; this is smaller than that observed in similar Schiff base ligands [20,21,22]. There are N–H···O intramolecular hydrogen-bonding interactions, which help stablilize this structure.

### 2.4. Thermal analysis

**Figure 2:**
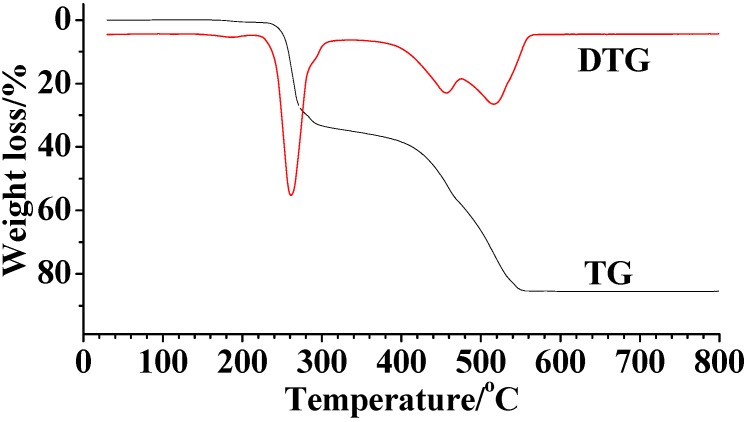
The TG-DTG curve of complex **1.**

The TG-DTG curves of the complexes are very similar and that of complex **1** is discussed as an example. The TG-DTG curves of complex **1** are shown in Figure 2. The DTG curves show mainly three stages in the decomposition process. The first stage decomposition temperature is in the range of 220.1 ^◦^C ~280.3 ^◦^C, with a mass loss of 29.56 %, which corresponds to the loss of two *p*-methyl-benzenes of a Schiff base ligand (calcd. 29.95 %). In the second to third stage of decomposition in the 285.3^◦^C ~560.6 ^◦^C temperature range, the remaining organic ligand molecule and two chloride ions are lost, with a mass loss of 56.90 % ( calcd. 57.07 %). The final product is the metal oxide Mn_2_O_3_ (13.54 %, calcd.12.97 %). These results are in good accordance with the composition of the complexes. 

### 2.5. Antibacterial activities

The *in vitro* antibacterial screening of the Schiff base ligand and its complexes dissolved in DMF has been carried out against three bacteria, viz., *Escherichia Coli, Staphylococcus Aureus* and *Bacillus Subtilis*, using a filter paper method [23] and an agar medium; the concentration of the test solutions was 5 mg·mL^-1^ (Table 3). Results showed that the antibacterial effects of these complexes were similar to those of the Zn and Mn complexes [12,13], which we had reported previously. 

**Table 3:** Data of antibacterial activity of ligand and complexes.

Compound	Diameter of inhibition zone (mm)		
	*Escherichia Coli*	*Staphylococcus Aureus*	*Bacillus Subtilis*
**L**	10.8	10.7	10.7
**1**	11.8	11.0	11.2
**2**	11.7	11.7	12.3
**3**	11.4	11.5	13.3
**4**	11.4	12.7	13.0
**5**	18.6	23.1	23.9

* Note: the data in the table are average values of three experiments, the diameter of the filter paper is 10 mm.

It is noteworthy that the complexes have higher activity than the corresponding free Schiff base ligand against the same bacteria, hence, one can conclude that complexation increases the antibacterical activity. On the other hand, the susceptibility of Schiff base ligand is almost the same for the three bacteria, while its complexes show some differences; the Cd(II) complex has the best antibacterial activities of all complexes against three bacterial spp. 

## 3. Experimental

### 3.1. General

MCl_2_·nH_2_O, *o*-vanillin, *p*-toluidine, and other chemical reagents were obtained from commercial sources and used without further purification. The metal contents were determined by EDTA complexometric titration after decomposition of a known amount of the complexes with concentrated nitric acid. Elemental analyses were carried out on an Elementar Vario EL III elemental analyzer. IR spectra on KBr pellets were recorded on a Nicolet NEXUS 670 FTIR spectrophotometer in the range of 4,000-400 cm^-1^. Molar conductivity of the complexes were measured with a Shanghai DDS-11A conductivity meter in methanol (1.0×10-3 mol·L^-1^). Thermal analyses were carried out using Mettler-Toledo TGA/SDTA851^e^ thermal analyzer with a heating rate of 10 ^◦^C·min^-1^ from 30 ^◦^C to 900 ^◦^C in an air atmosphere. 

### 3.2. Syntheses

**Figure 3:**
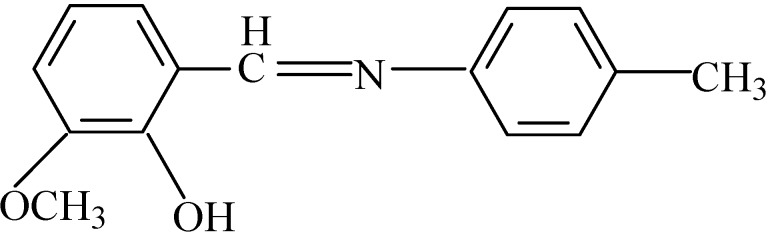
Structure of Schiff base ligand.

C_15_H_15_NO_2 _(**L**): Schiff base ligand (Figure 3) was prepared by the direct solution reaction, as reported in the literature [24], it was recrystallized in methanol before use.

MCl_2_L_2_: Preparation of MnL_2_Cl_2_ (**1**). The appropriate transition metal chloride salt (1 mmol) dissolved in anhydrous alcohol (10 mL) was stirred with Schiff base **L** (0.57 g, 2 mmol) in anhydrous alcohol (20 mL) and refluxed for 2 h in a water bath; after cooling to room temperature a solid deposited, which was washed with ethanol and dried. Yield 0.40 g (65%). Red single crystals of complex (**1**) suitable for X-ray diffraction were obtained from its mother liquid after slow evaporation at room temperature for five days.

### 3.3. Crystal structure determination

A single crystal of **1 **with approximate dimensions 0.32×0.26×0.08 mm^3^ was selected and coated with vaseline. Intensity data for the complex **1** was measured with a Rigaku R-AXIS RAPID diffractometer with graphite-monochromated Mo-Kα radiation (λ= 0.71073 Å ) at 296 K. Empirical absorption corrections were applied by use of the *ABSCOR* program. The structures were solved by direct methods and all calculations were performed with the aid of the SHELXL PC program [25]. The structures were refined by full-matrix, least-squares minimization of Σ(F_o_—F_c_)^2^ with anisotropic thermal parameters for all atoms except H atoms. The crystal data of the complexes **1 **was summarized in Table 4, selected bond lengths and angles in Table 5.

**Table 4:** Crystallographic Data for Complex **1.**

Empirical formula	C_30_H_30_Cl_2_MnN_2_O_4_	Density (g/cm^3^)	1.409
Formula weight	608.4	*Μ* (mm^-1^)	0.685
T/ K	293(2)	*F* (000)	1260
Crystal system	monoclinic	Max. and min. trans.	0.947 and 0.808
Space group	P2_1_/c	Data/restrains/parameters	6453 / 0 / 352
*a* (Å)	9.0111(18)	*θ* range /°	2.31 to 27.48
*b* (Å)	11.322(2)	Limiting indices	-11≤h≤11, -14≤k≤14, -36≤l≤36
*c* (Å)	28.130(6)	Reflections collected/ unique	11540 / 6453
*α* (°)	90	R_int_	0.0262
*β* (°)	92.29(3)	*GOOF* on *F*^2^	0.938
*γ* (°)	90	*R* and *wR* (*I > 2σ(I)*)	*R* = 0.0424, *wR* = 0.0984
*V* (Å^3^)	2867.6(10)	*R* indices (all data)	*R* = 0.0782, *wR* = 0.1076
*Z*	4	(Δρ)_max_, (Δρ)_min_ (e·Å^-3^)	0.504 and -0.504

**Table 5:** Selected Bond Lengths (Å) and Bond Angles (°) for Complex **1.**

Bond	Dist.	Bond	Dist.	Bond	Dist.
Mn(1)–O(3)	2.0771(15)	O(1)–C(16)	1.307(2)	O(4)–C(8)	1.420(3)
Mn(1)–O(1)	2.0885(15)	O(2)–C(21)	1.369(2)	N(1)–C(22)	1.308(3)
Mn(1)–Cl(2)	2.3921(9)	O(2)–C(23)	1.423(3)	N(1)–C(24)	1.418(3)
Mn(1)–Cl(1)	2.4233(8)	O(3)–C(1)	1.306(3)	N(2)–C(7)	1.302(3)
Mn(1)–O(2)	2.5229(16)	O(4)–C(6)	1.375(3)	N(2)–C(9)	1.411(3)
Mn(1)–O(4)	2.6036(17)				
Angle	(°)	Angle	(°)	Angle	(°)
O3–Mn(1)–O(1l)	146.55(6)	Cl(2)–Mn(1)–O(2)	158.05(4)	C(7)–N(2)–C(9)	128.51(19)
O3–Mn(1)–Cl(2)	103.21(5)	Cl(1)–Mn(1)–O(2)	84.43(5)	C(5)–C(6)–O(4)	126.2(2)
O1–Mn(1)–Cl(2)	91.32(5)	C(16)–O(1)–Mn(1)	123.39(13)	O(4)–C(6)–C(1)	112.9(2)
O3–Mn(1)–Cl(1)	91.55(5)	C(21)–O(2)–C(23)	118.34(18)	N(2)–C(7)–C(2)	123.3(2)
O1–Mn(1)–Cl(1)	111.66(5)	C(21)–O(2)–Mn(1)	109.75(12)	C(10)–C(9)–N(2)	117.6(2)
Cl(2)–Mn(1)–Cl(1)	110.27(3)	C(23)–O(2)–Mn(1)	125.46(15)	C(14)–C(9)–N(2)	122.7(2)
O3–Mn(1)–O(2)	92.23(6)	C(1)–O(3)–Mn(1)	124.31(14)	C(29)–C(24)–N(1)	123.57(19)
O1–Mn(1)–O(2)	67.63(5)	C(22)–N(1)–C(24)	128.11(18)	C(25)–C(24)–N(1)	116.89(19)

## 4. Supplementary Material

Supplementary crystallographic data have been deposited with the Cambridge Crystallographic Data Centre, CCDC No. 700722. Copies of this information may be obtained free of charge from The Director, CCDC, 12 Union Road, Cambridge CB2 1EZ, UK (Fax: + 44 1223 336033; Email: deposit@ccdc.cam.ac.uk or www: http://www.ccdc.cam.ac.uk).
